# 13109 Phosphorylation state of Myristoylated Alanine-Rich C-Kinase Substrate Effector Domain mimetics determines its cytotoxicity in glioblastoma and macrophage model

**DOI:** 10.1017/cts.2021.403

**Published:** 2021-03-30

**Authors:** Hasan Alrefai, Nicholas J. Eustace, Joshua C. Anderson, Patricia H. Hicks, Peter H. King, Christopher D. Willey

**Affiliations:** The University of Alabama at Birmingham School of Medicine

## Abstract

ABSTRACT IMPACT: This study provides insight into how MED2 impacts the immune cells surrounding glioblastoma that help it to grow and spread; having a more complete understanding of how MED2 works will help us better develop therapies that may one day enter the clinic to improve patient outcomes in glioblastoma. OBJECTIVES/GOALS: The purpose of this study was to determine whether the phosphorylation state of the MED2 peptide impacts its biological activity in GBM and macrophages. MED2 variants include the phosphorylatable wild-type (MED2), pseudo-phosphorylated (MED2-PP), non-phosphorylatable (MED2-NP) and control length (CTL2) peptides. METHODS/STUDY POPULATION: MED2, MED2-NP, MED2-PP, and CTL2 were screened against a panel of molecularly characterized glioblastoma patient derived xenografts and IL4/13 stimulated M2-like THP-1 macrophages. The luminescent cell viability assay, CellTiter-Glo, was used to determine viability. RESULTS/ANTICIPATED RESULTS: The proneural lines XD456 and X1441 were highly sensitive to 5 µM MED2 and 5 µM MED2NP compared to 5 µM MED2PP (p<0.001). There was no statistically significant difference between untreated, 5 µM CTL2, and 5 µM MED2PP groups or between the MED2NP and MED2 treated groups. M2-like THP-1 macrophages were highly sensitive to 10 µM MED2NP compared to 10 µM CTL2 (p<0.01) and 10 µM MED2PP (p<0.01) No statistically significant difference was observed between untreated, 10 µM MED2, 10 µM MED2PP, and 10 µM CTL2 groups. DISCUSSION/SIGNIFICANCE OF FINDINGS: The phosphorylation state of MED2 determines its toxicity. When MED2 is phosphorylated, it is nontoxic to GBM or M2-like macrophages. The non-phosphorylatable version is toxic to both GBM and M2-like macrophages. The wild-type peptide is toxic to GBM but not M2-like macrophages, suggesting that MED2 may be phosphorylated in M2-like macrophages.

